# Association of xenobiotic-metabolizing gene cytochrome P450 2E1 variants with preeclampsia in Chinese women

**DOI:** 10.1007/s43032-025-01890-y

**Published:** 2025-06-11

**Authors:** Kaifeng Hu, Qingqing Liu, Xinghui Liu, Huai Bai, Yujie Wu, Ping Fan

**Affiliations:** 1 Laboratory of Genetic Disease and Perinatal Medicine, Key Laboratory of Birth Defects and Related Diseases of Women and Children, Ministry of Education, West China Second University Hospital, Sichuan University, Chengdu, Sichuan China; 2 Department of Obstetrics and Gynecology, West China Second University Hospital, Sichuan University, Chengdu, Sichuan China

**Keywords:** Preeclampsia, CYP2E1, Genetic polymorphism, Oxidative stress, Xenobiotic metabolism

## Abstract

Environmental and genetic factors are related to the pathogenesis of preeclampsia (PE). Cytochrome P450 2E1 (*CYP2E1*) is crucial for the metabolism of endogenous and xenobiotic substances, possibly involved in the pathophysiology of PE. This study explored the association between *CYP2E1* 96-bp insertion/deletion (I/D) and rs2031920 (C-1054T) genetic variants and the risk of PE in 335 patients with PE and 1301 healthy pregnant women. The *CYP2E1* C-1054T variant was linked to an elevated risk of PE according to the dominant, genotype, and allele genetic models (*P* < 0.05). The genotype TT + CT remained a significant predictor of PE in the logistic regression model including age, gestational age, and body mass index at delivery (OR = 1.606, 95% CI: 1.137–2.286; *P* = 0.007). Moreover, the combined genotype TT + CT/II + ID of the C-1054T and 96-bp I/D variants further heightened the risk of PE, with the combined genotype DD/CC serving as the reference category (OR = 2.383, 95% CI: 1.381–4.106, *P* = 0.002). Furthermore, patients with the -1054T allele had lower serum albumin levels and total antioxidant capacity, and more severe proteinuria than those with the CC genotype (*P* < 0.05), and patients with the 96-bp I allele had a relatively higher atherosclerosis index than those with the DD genotype (*P* = 0.057). No significant differences in genotype frequencies of PE with severe features, platelet count, serum hepatic enzyme activities and creatinine levels were observed according to the different genotypes (*P* > 0.05). We conclude that the T allele of the C-1054T variant and its integration with the I allele of the 96-bp I/D variant in *CYP2E1* are linked to an elevated risk of PE in Chinese women.

## Introduction

Preeclampsia (PE) is a serious pregnancy complication marked by the onset of hypertension after 20 weeks of gestation, impacting multiple systems and organs [1]. PE affects 2%–8% of gestation women globally, representing a significant risk to both maternal and neonatal health [1, 2]. Although the etiopathogenesis of PE remains unclear, some studies have reported that PE is linked to inflammation, oxidative stress, and endothelial dysfunction and is probably the result of the interaction between detrimental environmental factors and disease susceptibility genes [2–8].

Cytochrome P450 2E1 (CYP2E1) is an important enzyme that metabolizes xenobiotics and endogenous substrates into various active intermediates, such as reactive oxygen species (ROS) and hepatotoxic or carcinogenic metabolites in the liver [9–12]. These highly-reactive intermediates are typically modified with hydrophilic chemical groups, converted into non-toxic and water-soluble metabolites, and excreted from the body. However, some intermediates can covalently bind to biological macromolecules, damage the function of these molecules, and contribute to cancer development, drug- or ethanol-induced liver impairment, and nonalcoholic fatty liver disease [10–12]. Genetic variation in *CYP2E1*, such as 96-bp insertion/deletion (I/D) and rs2031920 (C-1054 T, RsaI) in the 5′–fanking region, may affect the translation and activity of *CYP2E1* [13–15]. These two polymorphisms have been reported to be linked to higher risk of several cancer types [16–18], drug-related liver toxicity [19], polycystic ovary syndrome (PCOS) [9], gestational diabetes mellitus (GDM) [20], and unfavorable pregnancy outcomes [21].

Both environmental and genetic factors influence in the onset and progression of PE [2, 3, 6, 8, 22, 23]. Air pollutants, such as particulate matter (PM) 2.5, contain many harmful organic compounds metabolized and detoxified through xenobiotic metabolism in the liver [10, 24]. Epidemiological studies have suggested a relation between exposure of pregnant women to air pollutants and PE [23–25]. Notably, air pollutants are relatively prominent in the Sichuan Basin [26], and pregnant women in this area are more vulnerable to environmental pollutants. The active intermediates produced by CYP2E1 may impair biomacromolecule structure and function, contributing to cell dysfunction, oxidative stress, epigenetic changes, and apoptosis [10, 12, 27]. Therefore, CYP2E1 may get entangled in the pathophysiology of PE. However, there are limited studies on xenobiotic metabolism and xenobiotic-induced oxidative stress in PE, and it is unknown whether the 96-bp I/D and C-1054 T polymorphisms in *CYP2E1* are related to PE. Therefore, this study explored the association of these two polymorphisms in *CYP2E1* with PE susceptibility and estimate the impacts of the genetic variants on oxidative stress, clinical, and metabolic parameters in a Southwest Chinese women.

## Materials and Methods

### Study Participants

This case–control study involved 335 patients with PE and 1301 healthy pregnant women. The research was implemented at the Department of Obstetrics and Gynecology of West China Second University Hospital from 2006 to 2021. Written informed consent was obtained from each of these participants. The study accorded with the principles of the Declaration of Helsinki and was approved by the Institutional Review Board of the West China Second University Hospital of Sichuan University (2020–036 to Ping Fan).

The diagnosis of PE was based on the criteria of the American College of Obstetricians and Gynecologists (ACOG) Practice Bulletins for PE [1]: diastolic blood pressure (DBP) ≥ 90 mm Hg and/or systolic blood pressure (SBP) ≥ 140 mm Hg on two occasions ≥ 4 h apart after 20 weeks of pregnancy, and proteinuria ≥ 0.3 g/24 h or ≥ 2 + (dipstick method) or protein/creatinine ratio ≥ 0.3 mg/dL. In cases without proteinuria, new-onset hypertension with the new onset of any of the following: (i) platelet count < 100 × 10^9^/L; (ii) renal inadequacy: serum creatinine ≥ 1.1 mg/dL or a doubling of the serum creatinine levels without other renal disease; (iii) hepatic injury: liver transaminase levels elevated to twice the normal range; (iv) pulmonary edema; (v) visual symptoms or new-onset headache unresponsive to medication, unexplained by alternative diagnoses. Healthy controls were concurrently enrolled at the same hospital, with the inclusion criterion being a singleton pregnancy without complications.

The exclusion criteria for participants were twin/multiple pregnancies; chronic hypertension; pre-gestational diabetes; autoimmune disease; smoking; and renal, hepatic, cardiac, and other endocrine disorders. Control women further excluded other pregnancy complications, such as GDM, gestational hypertension, intrahepatic cholestasis of pregnancy, isolated fetal growth restriction, and premature deliveries, or women who had undergone in vitro fertilization.

Clinical variables including gestational age, maternal age, body mass index (BMI, kg/m^2^), SBP, DBP, the birth weight, height, and ponderal index (PI, g/cm^3^) of the neonates of these mothers, were recorded. Blood samples were collected after a minimum of 8 h of fasting in the third trimester of gestation or before childbirth, and serum or plasma samples were preserved at − 80 °C until analysis.

### Genotype Analysis

Genomic DNA was isolated from the peripheral blood leukocytes of the participants. The *CYP2E1* C-1054 T and 96-bp I/D genotypes were measured as previously described [9].

### Analysis of Complete Blood Count, Hepatic and Renal Function, Oxidative Stress, and Metabolic Indicators

White blood cell (WBC), red blood cell (RBC), and platelet counts and hemoglobin concentrations were measured using the fully automated hematology analyzer (XN-9100, Sysmex Corporation, USA). Serum alanine aminotransferase (ALT), aspartate aminotransferase (AST), and lactate dehydrogenase (LDH) activities (by kinetic method); total protein (by biuret method); albumin levels (by bromocresol green method); urea nitrogen (by urease method); and creatinine (by enzymatic method) were determined using the automatic chemiluminescence immunoassay analyzer (Atellica CH930, Siemens Healthineers, Germany). Fibrinogen concentrations were measured by coagulation method.

Serum apolipoprotein (apo)A1, apoB, high-density lipoprotein cholesterol (HDL-C), total cholesterol (TC), low-density lipoprotein cholesterol (LDL-C), triglyceride (TG), malondialdehyde (MDA), total antioxidant capacity (TAC), total oxidant status (TOS), atherosclerosis index (AI), and oxidative stress index (OSI) were measured as previously described [28–32]. The intra- and inter-assay variations for all determinations were less than 5% and 10%, respectively.

### Statistical Analysis

Data are shown as relative frequencies (%) or mean ± standard deviation (SD). Quantitative variables between the two groups/subgroups were compared using an independent sample *t*-test, non-parametric (Mann–Whitney U) test (for non-normally distributed variables), or analysis of covariance (to adjust for age, gestational age, and delivery BMI differences). The differences in percentages between the control and PE groups and the deviation of genotype distribution from Hardy–Weinberg equilibrium were assessed using the chi-squared (χ^2^) test. Logistic regression and χ^2^ analytical methods were used to calculate odds ratios (ORs) and 95% confidence intervals (CIs), evaluating the relative risk of PE associated with the two genetic variants. Statistical significance was set at a two-sided *P*-value < 0.05. All statistical analyses were performed using Statistical Program for Social Sciences (SPSS) 21.0.

Considering the T allele frequency of *CYP2E1* C-1054 T polymorphism and sample size, the power value was computed using a method previously described [22].

## Results

### Clinical Characteristics and Biochemical Parameters of the Participants

Table 1 shows that age and gestational age were significantly lower, but pre-pregnancy BMI and delivery BMI were significantly higher in the patients with PE than those in the control women. Additionally, SBP, DBP, ALT, AST, LDH, urea nitrogen, creatinine, TC, TG, apoB, apoB/apoA1 ratio, AI, TOS, MDA, and OSI were higher, but platelet count, serum total protein and albumin concentrations, albumin/globulin ratio, apoA1, HDL-C, as well as neonatal birth height, weight, and PI were lower in the PE group than in the control group after correcting for discrepancies in gestational age, maternal age, and BMI at delivery.

**Table 1:** Clinical and biochemical parameters of patients with PE and control women

	Controls (n = 1301)	PE (n = 335)	*P*	*P*^*a*^
**Clinical characteristics**				
Age (year)	34.31 ± 4.51	31.11 ± 6.33	< 0.001	
Pre-pregnancy BMI (kg/m^2^)	21.25 ± 2.69	22.52 ± 3.52	< 0.001	
Delivery BMI (kg/m^2^)	26.61 ± 2.78	28.04 ± 3.70	< 0.001	
Gestational age (wk)	39.19 ± 0.96	36.04 ± 3.37	< 0.001	
SBP (mmHg)	114.91 ± 10.03	154.29 ± 20.44	< 0.001	< 0.001
DBP (mmHg)	72.52 ± 8.08	99.20 ± 14.63	< 0.001	< 0.001
Neonatal birth height (cm)	49.85 ± 1.92	45.61 ± 5.93	< 0.001	< 0.001
Neonatal birth weight (g)	3376.40 ± 373.61	2645.25 ± 840.17	< 0.001	< 0.001
Neonatal PI (g/cm^3^)	2.74 ± 0.22	2.64 ± 0.82	0.404	0.001
**Complete blood count and hepatic and renal function**				
WBC (10^9^/L)	8.66 ± 2.05	9.49 ± 3.78	0.022	0.685
RBC (10^12^/L)	3.82 ± 0.36	3.91 ± 0.50	0.057	0.442
Hemoglobin (g/dL)	12.04 ± 1.00	12.12 ± 0.15	0.918	0.418
Platelet (10^9^/L)	172.90 ± 50.28	151.05 ± 62.87	< 0.001	< 0.001
ALT (U/L)	18.14 ± 15.71	66.51 ± 90.31	< 0.001	< 0.001
AST (U/L)	20.77 ± 9.91	55.69 ± 71.53	< 0.001	< 0.001
LDH (U/L)	175.19 ± 59.40	273.15 ± 165.48	< 0.001	< 0.001
Total protein (g/L)	64.68 ± 4.90	61.38 ± 6.78	< 0.001	< 0.001
Albumin (g/L)	37.01 ± 5.01	32.33 ± 7.03	< 0.001	< 0.001
A/G ratio	1.35 ± 0.37	1.15 ± 0.30	< 0.001	< 0.001
Urea nitrogen (mmol/L)	3.21 ± 2.43	6.37 ± 13.91	0.001	0.001
Creatinine (mg/dL)	0.50 ± 0.10	0.78 ± 0.41	0.001	0.001
Proteinuria (Dipstick method)*	**―**	2.18 ± 1.45		
Proteinuria (g/24 h)	**―**	3.43 ± 3.54		
Fibrinogen (μmol/L)	435.52 ± 73.58	449.04 ± 103.00	0.070	0.430
Metabolic parameters**				
TG (mmol/L)	3.62 ± 1.42	4.40 ± 1.98	< 0.001	< 0.001
TC (mmol/L)	6.03 ± 1.10	6.27 ± 1.85	0.068	0.002
HDL-C (mmol/L)	1.97 ± 0.42	1.62 ± 0.59	< 0.001	< 0.001
LDL-C (mmol/L)	3.16 ± 0.99	3.19 ± 1.21	0.728	0.790
AI	2.15 ± 0.66	3.13 ± 1.35	< 0.001	< 0.001
ApoA1 (g/L)	2.36 ± 0.44	2.10 ± 0.58	< 0.001	< 0.001
ApoB (g/L)	1.15 ± 0.26	1.31 ± 0.40	< 0.001	< 0.001
ApoB/apoA1	0.50 ± 0.15	0.66 ± 0.26	< 0.001	< 0.001
Oxidative stress parameters***				
TOS (μmol H_2_O_2_ Equiv./L)	21.07 ± 7.04	30.31 ± 13.70	< 0.001	< 0.001
TAC (mmol Trolox Equiv./L)	1.10 ± 0.19	1.07 ± 0.21	0.154	0.308
OSI	19.48 ± 7.33	26.21 ± 13.31	< 0.001	< 0.001
MDA (nmol/ml)	5.37 ± 1.22	5.58 ± 2.33	0.318	0.024

Values are presented as mean ± SD

*BMI*, body mass index; *DBP*, diastolic blood pressure; *SBP*, systolic blood pressure; *PI*, ponderal index; *WBC,* white blood cell; *RBC,* red blood cell; *ALT*, alanine aminotransferase; *AST*, aspartate aminotransferase; *LDH*, lactate dehydrogenase, *A/G ratio*, albumin/globulin ratio; T*G,* triglyceride; *TC*, total cholesterol; *HDL-C*, high-density lipoprotein cholesterol; *LDL-C*, low-density lipoprotein cholesterol; *AI*, atherosclerosis index; *apoA1*, apolipoprotein A1; *apoB*, apolipoprotein B; *TOS*, total oxidant status; *TAC*, total antioxidant capacity; *MDA*, malondialdehyde; *OSI,* oxidative stress index

^a^All comparisons of parameters were corrected for differences in age, gestational age, and delivery BMI between the two groups, except for the parameters of age, gestational age, and BMI

*Proteinuria (Dipstick method): “-” = 0, “ ± ” = 0.5, “1 + ” = 1, “2 + ” = 2, “3 + ” = 3, “4 + ” = 4

**Controls (*n* = 1092), PE (*n* = 231)

***Controls (*n* = 830), PE (*n* = 144)

### Genotypic and Allelic Frequencies of the CYP2E1 C-1054 T and 96bp-I/D Variants

The genotypic distributions of *CYP2E1* C-1054 T and 96 bp I/D conformed to Hardy–Weinberg equilibrium in the control and PE groups (all *P* > 0.05).

Table 2 shows that the frequencies of the TT + CT genotype (41.5% vs. 33.7%) and T allele (23.1% vs. 18.6%) of the C-1054 T variant were significantly higher in patients with PE than in the controls (OR = 1.397, 95% CI: 1.093–1.787, *P* = 0.008 for the dominant model; OR = 1.320, 95% CI: 1.076–1.621, *P* = 0.008 for the allele model). The TT + CT genotype remained a significant risk predictor of PE in a logistic regression model that included age, gestational age, and delivery BMI as covariates (OR = 1.606, 95% CI: 1.137–2.268, *P* = 0.007). The statistical power to detect an inheritance association was 0.978 for the C-1054 T genetic variation (significance level = 0.05; prevalence = 0.10). No statistical differences were observed between the control and PE participants across various genetic models for the 96-bp I/D variant (*P* > 0.05).

**Table 2:** Association of *CYP2E1* C-1054 T and 96-bp I/D polymorphisms with the risk of PE using different genetic models

	Controls (*n* = 1301)	PE (*n* = 335)	χ^2^	*P*
**C-1054 T**				
genotype				
CC	863 (66.3%)	196 (58.5%)		
CT	393 (30.2%)	123(36.7%)		
TT	45 (3.5%)	16 (4.8%)	7.339	0.025
Recessive				
TT	45 (3.5%)	16 (4.8%)		
CC + CT	1256 (96.5%)	319 (95.2%)	1.228	0.256
Dominant				
CC	863 (66.3%)	196 (58.5%)		
TT + CT	438 (33.7%)	139 (41.5%)	7.147	0.008*
Allele				
T	483 (18.6%)	155 (23.1%)		
C	2119 (81.4%)	515 (76.9%)	7.094	0.008**
**96-bpI/D**				
genotype				
DD	820 (63.0%)	200 (59.7%)		
ID	422 (32.4%)	120 (35.8%)		
II	59 (4.5%)	15 (4.5%)	1.396	0.498
Recessive				
II	59 (4.5%)	15 (4.5%)		
DD + ID	1242 (95.5%)	320 (95.5%)	0.002	0.964
Dominant				
DD	820 (63.0%)	200 (59.7%)		
II + ID	481 (37.0%)	135 (40.3%)	1.256	1.262
Allele				
I	540 (20.8%)	150 (22.4%)		
D	2062 (79.2%)	520 (77.6%)	0.856	0.335

Data are presented as number (%). Different genetic models (genotype, dominant, recessive, and allele models)

*Odds ratio (OR) = 1.397, 95% confidence interval (CI): 1.093–1.787

**OR = 1.320, 95% CI: 1.076–1.621

Table 3 shows the association of the C-1054 T and 96 bp-I/D genotype combinations in *CYP2E1* with PE. Due to the relatively small sample size of the −1054 TT and 96-bp II homozygotes, we combined them into heterozygous subgroups. The frequency of the combined genotype TT + CT/II + ID was higher in the PE group than in the control group (12.8% vs. 8.4%, *P* = 0.016). The TT + CT/DD and TT + CT/II + ID combined genotypes were further proven to be risk factors for PE when the combined genotype DD/CC (wild type) was set as a reference category in a multinomial logistic regression model (OR = 1.535, 95% CI: 1.010–2.334, *P* = 0.045; OR = 2.382, 95% CI: 1.381–4.106, *P* = 0.002, respectively).

**Table 3:** Frequencies of combined genotypes of *CYP2E1* C-1054 T and 96-bp I/D polymorphisms in patients with PE and control women

Genotype combinations	Controls (n = 1301)	PE (n = 335)	OR	95%CI	*P*
C-1054 T and 96-bp I/D*					
CC/DD	491 (37.7%)	104 (31.0%)	1.00	-	-
TT + CT/DD	329 (25.3%)	96 (28.7%)	1.535	1.010–2.334	0.045
CC/II + ID	372 (28.6%)	92 (27.5%)	1.240	0.809–1.901	0.324
TT + CT/II + ID	109 (8.4%)	43 (12.8%)	2.382	1.381–4.106	0.002

Data of genotype combinations are presented as number (%) of patients or controls

*Chi-squared test: χ^2^ = 10.265, *P* = 0.016. Odds ratio (OR) and 95% confidence interval (CI) were calculated in a multinomial logistic regression model including age, gestational age, and BMI at delivery as covariates, the DD/CC combined genotypes (wild-type) as the reference category

### Influences of the CYP2E1 C-1054 T and 96bp I/D Genotypes on Oxidative Stress, Clinical, and Metabolic Indexes

Table 4 shows that patients with the TT + CT genotype of the *CYP2E1* C-1054 T variant had lower TAC levels compared to those with the CC genotype (*P* = 0.019). The control women carrying the TT + CT genotypes had significantly higher BMI at delivery and lower apoB/apoA1 ratio (*P* < 0.05), and tended to have decreased SBP (*P* = 0.089), AI (*P* = 0.090), neonatal birth height (*P* = 0.065), and increased pre-pregnancy BMI (*P* = 0.067) than those carrying the CC genotype.

**Table 4:** Clinical, metabolic, and oxidative stress parameters according to *CYP2E1* C-1054 T and 96-bp I/D genotypes in patients with PE and control women

		**C-1054 T**				**96-bp I/D**			
		Control group		PE group		Control group		PE group	
		CC (n = 863)	TT + CT (n = 45 + 393)	CC (n = 196)	TT + CT (n = 16 + 123)	DD (n = 820)	II + ID (n = 59 + 422)	DD (n = 200)	II + ID (n = 15 + 120)
**Clinical characteristics**									
Age (years)	34.35 ± 4.53		34.24 ± 4.45	30.90 ± 6.84	31.41 ± 5.52	34.22 ± 4.59	34.47 ± 4.37	31.37 ± 6.33	30.73 ± 6.33
Pre-pregnancy BMI (kg/m^2^)	21.14 ± 2.72		21.47 ± 2.61	22.76 ± 3.39	22.22 ± 3.68	21.26 ± 2.77	21.23 ± 2.55	22.54 ± 3.59	22.49 ± 3.48
Delivery BMI (kg/m^2^)	26.49 ± 2.80		26.83 ± 2.75^a^	28.04 ± 3.53	28.03 ± 3.93	26.66 ± 2.82	26.51 ± 2.72	27.77 ± 3.63	28.44 ± 3.77
Gestational age (wk)	39.18 ± 0.99		39.20 ± 0.89	35.81 ± 3.52	36.35 ± 3.15	39.20 ± 0.94	39.17 ± 0.99	35.84 ± 3.33	36.32 ± 3.43
SBP (mmHg)	115.18 ± 10.05		114.38 ± 9.98	154.57 ± 21.18	153.89 ± 19.38	114.90 ± 10.01	114.93 ± 10.08	154.72 ± 21.37	153.69 ± 19.10
DBP (mmHg)	72.64 ± 7.84		72.30 ± 8.54	100.02 ± 14.73	98.01 ± 14.45	72.24 ± 8.33	73.07 ± 7.61^C^	99.26 ± 14.36	99.11 ± 15.06
Neonatal birth height (cm)	49.92 ± 1.99		49.71 ± 1.74	45.27 ± 6.13	46.09 ± 5.66	49.83 ± 2.00	49.89 ± 1.77	46.08 ± 6.22	44.96 ± 5.01
Neonatal birth weight (g)	3375.65 ± 377.52		3377.92 ± 366.16	2615.89 ± 805.62	2686.63 ± 889.22	3375.99 ± 376.02	3377.09 ± 369.96	2639.84 ± 867.78	2653.32 ± 801.60
Neonatal PI (g/cm^3^)	2.73 ± 0.22		2.74 ± 0.21	2.64 ± 0.74	2.65 ± 0.93	2.72 ± 0.21	2.77 ± 0.22	2.54 ± 0.90	2.77 ± 0.70
Metabolic parameters*									
TG (mmol/L)	3.60 ± 1.41		3.67 ± 1.43	4.53 ± 2.12	4.24 ± 1.79	3.62 ± 1.43	3.64 ± 1.40	4.35 ± 1.96	4.48 ± 2.03
TC (mmol/L)	6.06 ± 1.11		5.97 ± 1.07	6.14 ± 1.91	6.13 ± 1.78	6.03 ± 1.11	6.02 ± 1.08	6.39 ± 1.98	6.06 ± 1.61
HDL-C (mmol/L)	1.97 ± 0.43		1.97 ± 0.40	1.63 ± 0.56	1.60 ± 0.62	1.98 ± 0.43	1.96 ± 0.41	1.67 ± 0.58	1.52 ± 0.59
LDL-C (mmol/L)	3.18 ± 0.93		3.16 ± 1.07	3.26 ± 1.24	3.10 ± 1.18	3.15 ± 0.93	3.18 ± 1.08	3.23 ± 1.33	3.13 ± 0.98
AI	2.17 ± 0.71		2.10 ± 0.56	3.14 ± 1.35	3.13 ± 1.36	2.14 ± 0.62	2.16 ± 0.72	2.99 ± 1.19	3.38 ± 1.57
ApoA1 (g/L)	2.35 ± 0.47		2.38 ± 0.40	2.11 ± 0.54	2.10 ± 0.62	2.36 ± 0.46	2.37 ± 0.43	2.14 ± 0.59	2.05 ± 0.56
ApoB (g/L)	1.16 ± 0.26		1.13 ± 0.26	1.31 ± 0.40	1.31 ± 0.39	1.15 ± 0.26	1.14 ± 0.26	1.32 ± 0.42	1.30 ± 0.35
ApoB/apoA1	0.51 ± 0.16		0.49 ± 0.14^a^	0.65 ± 0.22	0.67 ± 0.31	0.50 ± 0.15	0.50 ± 0.16	0.65 ± 0.27	0.67 ± 0.25
Oxidative stress parameters**									
TOS (μmol H_2_O_2_ Equiv./L)	21.10 ± 6.91		21.01 ± 7.30	30.34 ± 14.50	30.27 ± 12.72	21.08 ± 7.07	21.06 ± 6.99	30.05 ± 13.09	30.83 ± 15.12
TAC (mmol Trolox Equiv./L)	1.10 ± 0.19		1.11 ± 0.20	1.11 ± 0.21	1.03 ± 0.21^b^	1.10 ± 0.20	1.11 ± 0.19	1.06 ± 0.20	1.10 ± 0.25
OSI	19.72 ± 7.24		19.02 ± 7.48	25.01 ± 11.74	27.84 ± 15.25	19.48 ± 7.49	19.48 ± 7.03	27.19 ± 14.44	24.28 ± 10.77
MDA (nmol/ml)	5.38 ± 1.23		5.36 ± 1.21	5.72 ± 2.22	5.41 ± 2.45	5.38 ± 1.20	5.35 ± 1.27	5.66 ± 2.47	5.38 ± 1.97

Values are presented as mean ± SD

*BMI*, body mass index; *DBP*, diastolic blood pressure; *SBP*, systolic blood pressure; *PI*, ponderal index; T*G,* triglyceride; *TC*, total cholesterol; *HDL-C*, high-density lipoprotein cholesterol; *LDL-C*, low-density lipoprotein cholesterol; *AI*, atherosclerosis index; *apoA1*, apolipoprotein A1; *apoB*, apolipoprotein B; *TOS*, total oxidant status; *TAC*, total antioxidant capacity; *MDA*, malondialdehyde; *OSI,* oxidative stress index

All comparisons of parameters were corrected for differences in age, gestational age, and delivery BMI between the two subgroups, except for the parameters of age, gestational age, and BMI

^a^*P* < 0.05: compared with the CC genotype subgroup in the control group

^b^*P* < 0.05: compared with the CC genotype subgroup in the PE group

^c^*P* = 0.048: compared with the DD genotype subgroup in the control group

*Controls (CC = 726, TT + CT = 35 + 331; DD = 683, II + ID = 50 + 359); PE (CC = 130, TT + CT = 15 + 86; DD = 143, II + ID = 9 + 79)

**Controls (CC = 552, TT + CT = 29 + 249; DD = 530, II + ID = 35 + 265); PE (GG = 78, TT + CT = 11 + 55; DD = 99, II + ID = 4 + 41)

Patients carrying the II + ID genotype of the *CYP2E1* 96 bp I/D variant tended to have increased AI (*P* = 0.057) and neonatal PI (*P* = 0.068) than those carrying the DD genotype (Table 4). The control women carrying the II + ID genotype had higher DBP (*P* = 0.048) and tended to have elevated neonatal PI (*P* = 0.077) than those carrying the DD genotype (Table 4).

### Impacts of the CYP2E1 C-1054 T and 96bp I/D Genotypes on Severity of PE, Blood Cell Count, Hepatic and Renal Function in Patients with PE

We further analyzed the effects of genotypes on PE severity, blood routine, and liver and kidney function in patients with PE. Based on ACOG guidelines [1], PE with severe features is defined as patients who have significant organ damage or with the new onset of any of the following: (i) systolic blood pressure ≥ 160 mm Hg and/or diastolic blood pressure ≥ 110 mm Hg; (ii) platelet count < 100 × 10^9^/L; (iii) serum creatinine concentrations ≥ 1.1 mg/dL; (iv) elevated blood concentrations of liver transaminases to twice normal concentration; (v) heart failure and/or pulmonary edema; (vi) new-onset headache unresponsive to medication and not accounted for by alternative diagnoses or visual symptoms.

Table 5 shows that patients carrying the genotype TT + CT of the *CYP2E1* C-1054 T polymorphism had significantly lower serum albumin (*P* = 0.001) and more severe proteinuria (Dipstick method, *P* = 0.009), and tended to have decreased albumin/globulin ratio (*P* = 0.058) compared with those carrying the CC genotype. No statistical differences in the indexes, including genotype frequencies of PE with severe features, WBC, RBC, hemoglobin, platelet, serum ALT, AST, LDH, total protein, urea nitrogen, creatinine, and fibrinogen levels, were observed between the CC and TT + CT genotypic subgroups, and between the DD and II + ID genotypic subgroups in patients (*P* > 0.05).

**Table 5:** Severity of PE, blood cell count, hepatic and renal function according to *CYP2E1* C-1054 T and 96-bp I/D genotypes in patients with PE

	C-1054 T genotype		96-bp I/D genotype	
	CC (*n* = 196)	TT + CT (*n* = 16 + 123)	DD (*n* = 200)	II + ID (*n* = 15 + 120)
PE subtypes*				
Without severe features, *n* (%)	29 (14.8%)	19 (13.7%)	29 (14.5%)	19 (14.1%)
With severe features, *n* (%)	167 (85.2%)	120 (86.3%)	171 (85.5%)	116 (85.9%)
WBC (10^9^/L)	9.82 ± 4.25	9.02 ± 2.93	9.65 ± 4.42	9.23 ± 2.35
RBC (10^12^/L)	3.95 ± 0.54	3.86 ± 0.43	3.90 ± 0.44	3.93 ± 0.59
Hemoglobin (g/dL)	12.16 ± 1.43	11.68 ± 2.12	11.97 ± 1.78	11.91 ± 1.75
Platelet (10^9^/L)	150.94 ± 65.75	151.27 ± 59.76	151.16 ± 60.59	150.94 ± 67.59
ALT (U/L)	72.08 ± 102.45	57.08 ± 77.12	67.86 ± 93.95	66.68 ± 87.75
AST (U/L)	47.58 ± 57.59	37.34 ± 27.18	46.78 ± 53.59	47.40 ± 61.60
LDH (U/L)	285.43 ± 184.04	252.82 ± 136.73	283.39 ± 177.86	253.37 ± 145.87
Total protein (g/L)	61.74 ± 6.55	60.84 ± 7.18	61.40 ± 7.56	61.34 ± 5.22
Albumin (g/L)	33.74 ± 6.87	31.26 ± 6.60^a^	31.95 ± 7.19	32.83 ± 6.45
A/G ratio	1.20 ± 0.30	1.13 ± 0.29	1.14 ± 0.28	1.17 ± 0.31
Urea nitrogen (mmol/L)	7.09 ± 17.95	5.61 ± 6.12	7.40 ± 17.51	4.77 ± 2.78
Creatinine (mg/dL)	0.80 ± 0.44	0.75 ± 0.39	0.79 ± 0.40	0.77 ± 0.45
Proteinuria (Dipstick method)**	1.89 ± 1.43	2.31 ± 1.40^a^	2.09 ± 1.45	2.04 ± 1.40
Proteinuria (g/24 h)	3.29 ± 3.16	3.80 ± 4.04	3.41 ± 3.70	3.46 ± 3.44
Fibrinogen (μmol/L)	450.03 ± 107.29	446.32 ± 93.35	443.60 ± 98.63	456.99 ± 106.61

Values are presented as mean ± SD or n (%)

*PE,* preeclampsia; *WBC,* white blood cell; *RBC,* red blood cell; *ALT*, alanine aminotransferase; *AST*, aspartate aminotransferase; *LDH*, lactate dehydrogenase, *A/G ratio*, albumin/globulin ratio

*Chi-squared test: χ^*2*^ = 0.084, *P* = 0.772 between the CC and TT + CT genotypic subgroups; χ^*2*^ = 0.012, *P* = 0.913 between the DD and II + ID genotypic subgroups

**Proteinuria (Dipstick method): “-” = 0, “ ± ” = 0.5, “1 + ” = 1, “2 + ” = 2, “3 + ” = 3, “4 + ” = 4

All comparisons of quantitative parameters were corrected for differences in age, gestational age, and delivery BMI between the two subgroups

^a^*P* < 0.05: compared with the CC genotype subgroup

## Discussion

As far as we know, this is the first study to confirm that the C-1054 T variant of *CYP2E1*, rather than the 96-bp I/D variant, is linked to a higher risk of PE in the Chinese women. We also demonstrated that the TT + CT/II + ID combined genotype is associated with the risk of PE when the DD/CC combined genotype was set as a reference. Furthermore, we observed that patients carrying the CT + TT genotype had lower serum albumin and TAC levels, and more severe proteinuria compared with those carrying the CC genotype. Patients carrying the II + ID genotype tended to have increased AI and neonatal PI compared with those carrying the DD genotype, indicating that these two variants of the xenobiotic-metabolizing gene may be involved in oxidative stress, lipid metabolism, serum albumin levels, proteinuria progression, and fetal development in patients.

Xenobiotic metabolism is an important pathway through which the body metabolizes environmental organic pollutants [10]. CYP2E1 is one of the essential xenobiotic metabolism-related enzymes in the liver and is involved in the biotransformation of more than 80 xenobiotics [10, 19]. Intermediates produced by CYP2E1, including ROS and other harmful components, may lead to oxidative stress and cellular dysfunction [10, 12, 19, 27]. Oxidative stress and environmental pollutants are risk factors for PE [2, 22–25, 28, 33]. The expression and enzymatic activity of CYP2E1 are impacted by genetic variants, disease status, and environmental factors [10, 16]. Therefore, probing *CYP2E1* genetic variants in women with PE may aid in identifying genetic predispositions and clarify the etiology and pathogenesis of PE.

Distribution of the *CYP2E1* C-1054 T polymorphism showed obvious ethnic differences. The frequency of the variant allele (T allele, RsaI c2) varies from 17.7% to 25% in the East Asians [9, 10, 18] and is higher compared to that in Caucasians (4.0%) [10]. The T allele raises the transcriptional activation of the *CYP2E1* by affecting transcription factor binding [14]. This allele is related to increased risk of hepatitis B-related hepatocellular carcinoma [34], colorectal cancer in Brazilians [17], PCOS, and GDM in Chinese women [9, 20]. It also protects against lung cancer [11], bladder cancer [35], and drug-related hepatotoxicity [19]. In addition, the T allele is linked to lower birth weights of neonates whose mothers were exposed to prenatal disinfection by-products [21]. Herein, the frequency of the T allele of the *CYP2E1 C-1054 T* polymorphism was 18.6% in the control group and 23.1% in the PE group. We demonstrated that the T allele is a genetic risk factor for PE in the Chinese women. Additionally, we observed that patients with PE had significantly higher TOS, OSI, and MDA levels compared with the healthy pregnant women, and patients carrying the CT + TT genotype had lower serum albumin concentrations and TAC levels, and worse proteinuria compared with those carrying the CC genotype. However, in this study, we did not observe that this genetic variant was associated with the severe features of PE such as platelet count, hepatic enzyme activities, and serum creatinine levels. Our finding suggests that the T allele of the C-1054 T polymorphism may affect the redox status of the body due to the reduced antioxidant capacity and be related to lower serum albumin levels and more severe proteinuria in the patients.

The 96-bp insertion variant (I allele) of the *CYP2E1* 96-bp I/D up-regulates the transcriptional activity of *CYP2E1* [13]. The I allelic frequency of the 96-bp variation is relatively high in Asians (15 − 23.7%) but comparatively low in Caucasians (2%) and African-Americans (10%) [9, 13, 18]. Several studies have shown that carriers of the 96-bp I allele or II homozygote are linked to an increased risk of colorectal cancer and drug-induced hepatic damage [18, 19]. In this research, the 96-bp I allelic frequency was 20.8% in the control women and 22.4% in the PE patients. No statistical discrepancies were observed between the control and PE groups based on the various genetic models for the *CYP2E1* 96-bp I/D variant. No significant differences in genotype frequencies of PE with severe features, platelet count, proteinuria, hepatic enzyme activities, serum protein and creatinine levels were observed between the II + ID and DD genotypic subgroups. Nevertheless, we observed that patients with the genotype II + ID had a relatively high AI than those with the DD genotype. It is well known that gestation is a stress status associated with atherogenic lipid formation in women [36, 37]. Consistent with previous reports [22, 28], in this study, we indicated that women with PE had significantly higher TC, TG, apoB, apoB/apoA1 ratio, and AI, but lower HDL-C levels compared to the healthy pregnant women. Our study suggests that the 96-bp insertion allele may contribute to the atherogenic lipid profile in patients. Moreover, we certified that the combined genotype TT + CT/II + ID of the C-1054 T and 96-bp I/D polymorphisms in *CYP2E1* further heightened the risk of PE compared to the reference combined genotype CC/DD. Additional research is needed to clarify this issue and uncover the underlying mechanisms.

This study had some limitations. First, we could not analyze the −1054 TT and 96-bp II genotypes as separate subgroups owing to the comparatively low frequencies of minor allelic homozygosity. A larger sample size is needed to estimate dose-dependent genotypic features. Second, we could not determine the activity or protein levels of CYP2E1. Further measurements of enzyme activities or levels might be beneficial for clarifying the correlation between the risk of PE and genetic variations. Third, due to hemolysis or insufficient sample volume, we could not measure metabolic and oxidative stress parameters in some participants, which might have led to failure to achieve statistical significance or affected the power of these indices. Fourth, we could not analyze clinical features of severe PE, such as the frequencies of pulmonary edema, etc. Further analysis of these features will help us better understand the mechanism of association between the *CYP2E1* C-1054 T and 96-bp I/D genetic variants and the development of PE.

In conclusion, we demonstrated that the T allele of the C-1054 T variant and the TT + CT/DD and TT + CT/II + ID combined genotypes of the C-1054 T and 96-bp I/D polymorphisms in *CYP2E1* were associated with increased risk of PE in the Chinese population. Additionally, we showed that patients with the CT + TT genotype had decreased serum albumin and TAC levels and increased proteinuria, and those with the genotype II + ID had an unfavorable lipid profile. Our results provide a novel evidence that genetic variants in enzymes related to xenobiotic metabolism may be involved in the pathogenesis of PE.

## Data Availability

The datasets used and/or analyzed during the current study are available from the corresponding author on reasonable request.
